# Anti-Melanogenic Activities of *Sargassum fusiforme* Polyphenol-Rich Extract on α-MSH-Stimulated B16F10 Cells via PI3K/Akt and MAPK/ERK Pathways

**DOI:** 10.3390/foods13223556

**Published:** 2024-11-07

**Authors:** Bei Chen, Honghong Chen, Kun Qiao, Min Xu, Jingna Wu, Yongchang Su, Yan Shi, Lina Ke, Zhiyu Liu, Qin Wang

**Affiliations:** 1 School of Life Sciences, Xiamen University, Xiamen 361102, China; chenbeifjfri@foxmail.com (B.C.); chenhh6@mail.sustech.edu.cn (H.C.); yshi@xmu.edu.cn (Y.S.); kerlinda@xmu.edu.cn (L.K.); 2 Fisheries Research Institute of Fujian, Key Laboratory of Cultivation and High-Value Utilization of Marine Organisms in Fujian Province, Xiamen 361013, China; qiaokun@xmu.edu.cn (K.Q.); xumin1315@foxmail.com (M.X.); suyongchang@stu.hqu.edu.cn (Y.S.); 3 School of Environmental Science and Engineering, Southern University of Science and Technology, Shenzhen 518055, China; 4 Department of Pharmacy, Xiamen Medical College, Xiamen 361023, China; wjn@xmmc.edu.cn

**Keywords:** *Sargassum fusiforme*, polyphenol extracts, tyrosinase, anti-melanogenesis

## Abstract

Background: Melanin overproduction leads to pigmented skin diseases. Brown algae polyphenols, non-toxic secondary metabolites, exhibit potential bioactivities. *Sargassum fusiforme*, an edible seaweed, has been underexplored in the field of beauty despite its polyphenol richness. Methods: Polyphenols from *S. fusiforme* were extracted using macroporous resin (SFRP) and ethyl acetate (SFEP). Their antioxidant and anti-aging properties, tyrosinase inhibitory activities, and mechanisms were assessed. The melanogenesis inhibition effect and mechanism by SFRP was examined in B16F10 melanoma cells. Results: Both SFRP and SFEP demonstrated scavenging activities against DPPH, superoxide anion, and hydroxyl radicals. SFRP showed stronger anti-collagenase and anti-elastase effects. They dose-dependently inhibited mushroom tyrosinase, with IC50 values of 9.89 μg/mL for SFRP and 0.99 μg/mL for SFEP. SFRP reversibly inhibited tyrosinase, while SFEP showed irreversible inhibition. SFRP also suppressed melanin content and intracellular tyrosinase activity in B16F10 cells, downregulating the expression of microphthalmia-associated transcription factor, tyrosinase, and tyrosinase-related protein 1 and 2 expression through the PI3K/Akt and MAPK/ERK signal pathways. Conclusions: *S. fusiforme* polyphenols, especially SFRP, exhibit promising antioxidant, anti-aging, and melanogenesis inhibitory properties, highlighting their potential application as novel anti-melanogenic agents in cosmetics and the food industry.

## 1. Introduction

Melanin is the primary determinant of human skin, eye, and hair color, and it serves as an essential protectant against the harmful effects of ultraviolet radiation (UV) [1]. However, irregular pigmentation is considered a type of skin pigmentation disorder, including chloasma, freckles, vitiligo, moles, and leukoderma, which pose significant esthetic concerns for individuals seeking a flawless skin tone [2,3]. Melanogenesis is a complex process involving a series of reactions and multiple melanogenic enzymes. Firstly, L-tyrosine is hydroxylated by tyrosinase (TYR) to form L-dihydroxyphenilalanine (L-DOPA), which is subsequently oxidized into dopaquinone. And then highly reactive dopaquinone can spontaneously polymerize to form DOPA chrome, and tyrosine-related protein 2 (TRP2) converts DOPA chrome to 5,6-dihydroxyindole-2-carboxylic acid (DHICA) or 5,6-dihydroxyindole (DHI). Finally, DHICA and DHI are polymerized with the intermediates, eventually leading to melanin production [4]. TYR serves as the pivotal regulatory enzyme in melanin metabolism, with aberrant TYR expression potentially causing pigmentary disruptions. For instance, vitiligo typifies a hypopigmentation condition associated with diminished pigment or TYR function. In contrast, freckles represent a benign hyperpigmentation common among Asian women, attributed to heightened melanin deposition [5]. Common skin-lightening agents, such as kojic acid (KA), hydroquinone, and arbutin, which are effective in suppressing TYR activity, may induce adverse reactions including pigmented contact dermatitis, genotoxicity, and potential carcinogenicity [6,7]. The exploration of non-toxic, natural compounds for the treatment of skin pigmentation disorders has become a predominant research focus.

Polyphenolic compounds in brown algae have been found to possess a wide range of physiological properties including antioxidant, anti-tumor, anti-inflammatory, anti-allergic, and anti-microbial activities [8,9,10,11]. Due to its extensive biological activities and natural non-toxicity, brown algae polyphenolic compounds have attracted significant attention from researchers recently. It has been found to be useful as a potential hyperpigmentation agent in the development of skin-whitening substances. For example, phloroglucinol, eckol, and dieckol isolated from *Ecklonia cava* and 974-A, and phlorofucofuroeckol-A and eckol isolated from *Ecklonia stolonifera* significantly inhibited the activity of mushroom TYR (mTYR) and melanin synthesis in B16F10 cells [12,13]. Dioxinodehydroeckol isolated from *Ecklonia stolonifera* decreased the expression of TYR, TRP1, and TRP2 through the transcriptional downregulation of the microphthalmia-associated transcription factor (MITF) via PI3K/Akt-mediated signaling pathway in α-MSH-stimulated B16F10 cells [14]. Jang et al. showed that 4-hydroxyphenethyl, derived from *Hizikia fusiformis*, remarkably alleviated the hyperpigmented spots in brown guinea-pig induced by UVB, through inhibited the activity of mTYR and decreased the content of melanin [15].

*Sargassum fusiforme* is an economically significant seaweed along the coast of China, recognized for its high nutritional content. Utilized as both a food and natural medicine in China, Japan, North Korea, and South Korea, *S. fusiforme* has recently made notable advancements in industries related to natural pharmaceuticals, nutraceuticals, and cosmetics. This progress is attributed to its array of health benefits, including antioxidant, anti-inflammatory, antitumor, antidiabetic, immunomodulatory, antiviral, antibacterial, and anticoagulant properties [16]. In the field of cosmetics and nutricosmetics, there is currently a lack of research on the melanin inhibitory activity of *S. fusiforme* extract. The aim of this study was to enrich the phenolic compounds in *S. fusiforme* by macroporous resin or ethyl acetate, and evaluate and compare the antioxidant activity, anti-wrinkle activity, and whitening activity of the two and deeply investigate the mechanism of melanogenesis inhibition using mouse B16F10 cells, which could lead to the development of novel skincare products or dietary supplements for skin whitening.

## 2. Materials and Methods

### 2.1. Materials

Human leukocyte elastase (E8140), N-[3-(2-furyl)a-cryloyl]-Leu-Gly-Pro-Ala (FALGPA) (F5135), N-(methoxysuccinyl)-Ala-Ala-Pro-Val-4-nitroanilide (MAAPVN) (M4765), collagenase from *Clostridium histolyticum* (C0130), mTYR, 3,4-dihydroxyphenylalanine (L-DOPA), L-tyrosine (L-TYR), and phenazine methosulfate (PMS) were purchased from Sigma-Aldrich (Sigma-Aldrich Corp., St. Louis, MO, USA). A PierceTM BCA Protein Assay Kit and Trypsin-EDTA Solution was purchased from Thermofisher (Thermo Fisher Scientific Inc., Waltham, MA, USA). A CellTiter 96 Aqueous Non-Radioactive Cell proliferation Assay kit was purchased from Promega (Promega Corp., Madison, WI, USA). High-Efficiency RIPA tissue/cell rapid lysis solution purchased from Solarbio Science & Technology (Beijing Solarbio Science & Technology Co., Ltd., Beijing, China). Antibody TYR (NBP2-67233) was purchased from Novus Biologicals (Novus Biologicals Inc., Littleton, CO, USA), and Antibody TRP1 (ab235447), TRP2 (ab221144), and MITF (ab140606) were obtained from Abcam (Abcam Ltd., Cambridge, MA, USA). Antibody AKT (#9272), p44/42 MAPK (ERK1/2) (#4695), Phospho-p44/42 MAPK (ERK1/2) (#4370) and Phospho-AKT (#9271T) were purchased from Cell Signaling Technology (Cell Signaling Technology Inc., Beverly, MA, USA). Antibody GADPH (6004-1-lg) was purchased from Proteintech (Proteintech Group Inc., Wuhan, China).

### 2.2. Preparation of S. fusiforme Polyphenols

The *S. fusiforme* was harvested from Dongtou Island, Zhejiang Province, in April 2018. The sample was washed clean, and then freeze-dried. The dried *S. fusiforme* sample was collected to grind powder with liquid nitrogen. Following a 100-mesh sieve process, the powder was double-extracted using a 40% ethanol/water solution at a solid-to-liquid ratio of 1:15. This extraction occurred over 60 min at 70 °C, with intermittent stirring every 10 min. The resulting extract was subsequently filtered and centrifuged at 12,000 rpm for 30 min. The supernatants were then gathered, and ethanol was evaporated using a rotary evaporator. The crude *S. fusiforme* extract (SFC) underwent further centrifugation at 12,000 rpm for an additional 30 min, followed by filtration through a 0.45 μM filter to obtain the final product.

SFC was further purified with XDA-7 macroporous adsorption resin to obtain *S. fusiforme* polyphenol extract. Briefly, the XDA-7 resin was soaked in 95% ethanol for 24 h to make it expand fully, and then the resin was washed thoroughly with deionized water to remove all ethanol. Next, the resin was treated with 5% HCl and 4% NaOH solutions for 8 h, respectively, to remove impurities in the resin. Before loading the extract, the chromatographic column (ND8/DB08 Ø: 10 mm; h: 300 mm) containing XDA-7 resin was equilibrated with water at a flow rate of 1 mL/min for 3 BV. Next, the crude extract were loading into the resin with a flow rate of 0.5 mL/min. After the sample was adsorbed, the loaded resin was washed with deionized water at a flow rate of 1 mL/min with 1 BV. The XDA-7 microporous resin, loaded with polyphenol, was eluted using 60% ethanol solutions at a flow rate of 2 mL/min for 3 BV, and the resulting eluent was subsequently concentrated with a Buchi rotary evaporator to eliminate ethanol. Ultimately, the concentrate was freeze-dried in a vacuum to obtain the power of purified *S. fusiforme* polyphenol resin extract (SFRP).

SFC underwent triple extraction with trichloromethane, each time preserving the aqueous phase. Subsequently, ethyl acetate was used for further triple extractions, and the collected ethyl acetate fractions yielded the *S. fusiformie* polyphenol ethyl acetate fraction (SFEP). After freeze-drying, the powder was sealed and stored at −80 °C.

The total polyphenol content (TPC) was assessed by the colorimetric Folin–Ciocalteu method with slight modifications [17].

### 2.3. Evaluation of Antioxidant Activity

The scavenging abilities for hydroxyl and superoxide anion radicals were assessed using reagent kits from Nanjing Jiancheng Bioengineering Institute, following the manufacturer’s instructions. The scavenging activity against 1-diphenyl-2-pycryl-hydrazyl (DPPH) radicals was determined according to methods previously described in the literature [18].

### 2.4. Assessing the Inhibitory Effects of Elastase and Collagenase

The inhibitory effects of SFRP and SFEP on elastase and collagenase were assessed as previous studies described [19]. Epigallocatechin gallate (EGCG) at a concentration of 0.1 mg/mL was utilized as a positive control.

### 2.5. Assessment of mTYR Activity

The enzyme activity was assessed according to a previous study with slight modifications [20]. Briefly, the activities of mTYR were measured using L-DOPA as the substrate, respectively. The reaction system (3 mL) contained 0.5 mM L-DOPA in 50 mM Na_2_HPO_4_-NaH_2_PO_4_ buffer (pH 6.8), and different concentrations of SFRP and SFEP. All solutions were preheated at 30 °C in advance. The enzyme activity was determined by measuring the optical density at 475 nm (ε = 3700 M^−1^cm^−1^) accompanying the oxidation of L-DOPA each 5 s for 10 min using a Thermo MULTISKAN GO spectrophotometer. The degree of inhibition by the addition of SFRP and SFEP was exhibited as the percentage required to achieve a 50% loss of activity (IC50). The inhibition mechanism on the mTYR was determined following different concentrations of SFRP and SFEP, by controlling the concentration of substrate L-DOPA unchanged and changing the concentration of mTYR.

### 2.6. Cell Culture

The B16F10 mouse melanoma cell line, obtained from the Academy of Sciences cell bank in Kunming, China, was maintained in DMEM medium (Gibco Life Technologies, Paris, France) supplemented with 10% (*v*/*v*) fetal bovine serum (FBS) and 1% penicillin/streptomycin at 37 °C and 5% CO_2_.

### 2.7. Cell Viability Measurement

The cell viability was measured using the CellTiter 96 AQueous Non-Radioactive Cell Proliferation assay according to the manufacturer’s instructions to evaluate the effect of SFRP on B16F10 cells. Briefly, the SFRP were co-incubated with B16F10 cells for 24 h, and then MTS solution was added for 2 h. Absorbance at 490 nm was assessed using a microplate reader (Tecan, Morrisville, NC, USA), with cell viability computed against the control.

### 2.8. Measurement of Melanin Contents in Mouse B16F10 Cells

The intracellular melanin contents were evaluated according to the previous study with slight modifications [21]. In brief, B16F10 cells were seeded in 6-well plates at a density of 1.5 × 10^5^ cells/well. After the cells attached overnight, different concentrations of SFRP (0, 12.5, 25, 50, and 100 μg/mL) were supplemented to individual wells, with 100 nM α-MSH for 60 h, respectively. And then, the medium was removed and cells were washed twice with ice-cold phosphate-buffered solution (PBS). The cell pellet was dissolved in 1M NaOH at 95 °C for 30 min to quantify melanin content at 405 nm. Melanin levels were calculated as percentage changes relative to the control group, normalized to 100%.

### 2.9. Determination of Relative TYR Activity in Mouse B16F10 Cells

The specific intracellular TYR activity was conducted according to a previous study [21]. The cells were incubated with different concentrations of SFRP and α-MSH for 60 h. The cell pellet was collected and resuspended with 1% Triton X-100 solution containing 1 mM PMSF. The cells were frozen and thawed three times to make the cell disruption thorough. And then, the cell lysate was centrifuged at 12,000 rpm for 10 min and the supernatants were collected to measure the TYR activity. The amount of protein was measured using the PierceTM BCA Protein Assay Kit (Thermo Fisher Scientific Inc., Waltham, MA, USA) and was homogenized. An amount of 50 μL enzyme solution was incubated with 150 μL 0.5 mM L-DOPA at 37 °C for 30 min. The optical densities (ODs) of the mixture were detected at 475 nm in a microplate reader to determine the activity of intracellular TYR.

### 2.10. Western Blotting

B16F10 melanoma cells were seeded in a 6-well plate at 1.5 × 10^5^ cells/well and treated with different concentrations of SFRP and α-MSH for 60 h. The cells were centrifuged at 4 °C for 30 min to obtain the cell pallet, and then the cell pallet was resuspended with RIPA tissue/cell rapid lysis solution and incubated on ice for 1h. Next, the cell lysate was centrifuged at 12,000 rpm for 30 min at 4 °C and the supernatants were collected. Protein content and protein blotting assays were conducted based on previous research [22].

### 2.11. In Vitro Skin Irritation Test

The in vitro skin irritation test followed the Organization for Economic Co-operation and Development (OECD TG439) guidelines and the EpiSkin™ standard operating procedures. The specific method was conducted according to a previous study [23]. Briefly, epidermis units were preincubated at 37 °C, 5% CO_2_ for 24 h. After preincubation, they were exposed to 10 μL SFRP (1, 10, 100 mg/mL), 5% SDS, or H_2_O for 15 min at room temperature. Treated units washed with 25 mL PBS and wiped with cotton bud. Tissue units were moved into fresh medium and incubated for 42 h, followed by placing the units in wells filled with MTT at a concentration of 0.3 mg/mL. Tissue units were removed, with epidermis separated, and placed into microtubes with 500 μL acidic isopropanol. Tubes were stored for 4 h at room temperature in dark and vortexed during incubation for extraction. Two wells per tissue were transferred to a 96-well plate; absorbance at 570 nm was read on a microplate reader.

### 2.12. Statistical Analyses

Statistical analyses were conducted with GraphPad Prism 8.2.0 (Graphpad Software Inc., La Jolla, CA, USA) and SPSS 26.0 software platforms, presenting results as mean ± SD. Variance homogeneity was ensured. Differences between the groups were examined using one-way ANOVA, and multiple comparisons were evaluated by Tukey or LSD HSD test, with *p* < 0.05 indicating significance.

## 3. Results

### 3.1. Extraction and Preparation of S. fusiforme Polyphenol Extracts

In this study, *S. fusiforme* was extracted twice using 40% ethanol. The extract was then vacuum-rotary evaporated and freeze-dried to obtain the crude polyphenol extract SFC. The extract was further purified using macroporous adsorption resin XDA-7, yielding the purified polyphenol SFRP. Additionally, the extract was subjected to repeated equal volume extractions with ethyl acetate three times, resulting in the ethyl acetate phase polyphenol SFEP. The polyphenol content in SFC, SFRP, and SFEP was determined using the Folin-phenol method. The crude extract SFC contained 4.6% polyphenols. After purification with the macroporous resin and ethyl acetate, the polyphenol concentration increased more than tenfold compared to the crude extract. Notably, the polyphenol content in SFRP reached 56.7%, and SFEP contained 44.75% polyphenols.

### 3.2. In Vitro Antioxidant Capacity of S. fusiforme Polyphenol Extracts

**Figure 1:**
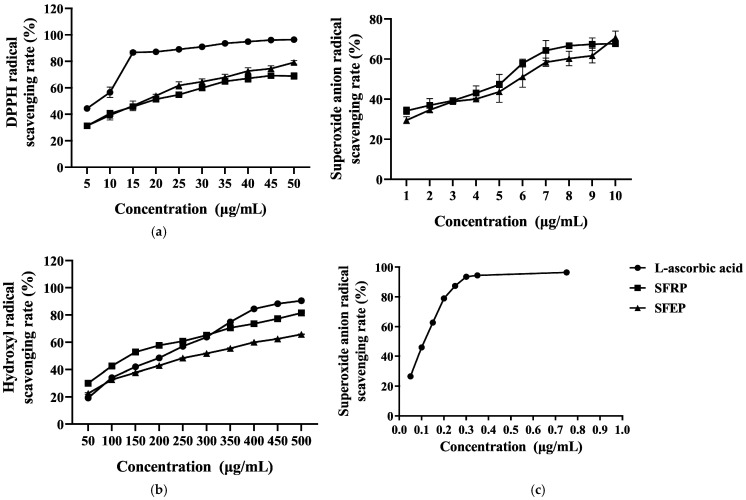
In vitro antioxidant capacity of *S. fusiforme* polyphenol extracts. (**a**) DPPH radical, (**b**) hydroxyl radicals and (**c**) superoxide anion radical scavenging activity of SFRP and SFEP in comparison to L-ascorbic acid. Each data point represents the mean ± standard deviation (SD) of n = 4 experimental replicates.

To evaluate the antioxidant activity of polyphenols from marine algae, this study used L-ascorbic acid as a control and measured the free radical scavenging abilities of SFRP and SFEP against DPPH, superoxide anion, and hydroxyl radicals. As shown in Figure 1a, the scavenging rates of DPPH by SFRP and SFEP gradually increased with the sample concentration. At a concentration of 50 μg/mL, the scavenging rates of DPPH by SFRP and SFEP reached 68.98% and 75.19%, respectively. The IC50 values for SFRP and SFEP were 16.86 and 15.01 μg/mL, respectively.

Reactive oxygen species (ROS) in organisms include superoxide anion, hydroxyl radicals, etc. When these ROS accumulate excessively in the body, they can react with biomacromolecules such as DNA, proteins, and lipids, damaging cell structures. The inhibitory effects of SFRP and SFEP on hydroxyl radicals are shown in Figure 1b. SFRP exhibited the strongest inhibitory effect on hydroxyl radicals, with an inhibition rate of 81.56% at 500 μg/mL. Within the concentration range of 50–300 μg/mL, the inhibitory effect of SFRP on hydroxyl radicals was stronger than that of L-ascorbic acid.

As shown in Figure 1c, L-ascorbic acid has a strong inhibitory effect on superoxide anion radicals, with an inhibition rate of 96.42% at 0.75 mg/mL. SFRP and SFEP also show a certain inhibitory effect on superoxide anion radicals, exhibiting a dose–effect relationship with sample concentration, though generally less potent than L-ascorbic acid. Among them, SFRP exhibits a stronger inhibitory effect on superoxide anion radicals than SFEP.

### 3.3. In Vitro Skin Anti-Aging Activity of S. fusiforme Polyphenol Extracts

**Figure 2:**
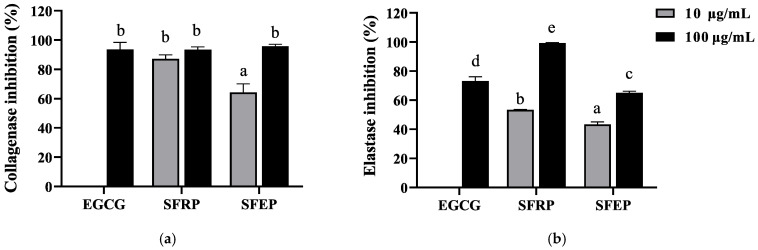
Inhibition of collagenase (**a**) and elastase (**b**) by polyphenol extracts of *S. fusiforme*. Data are expressed as the mean ± SD from *n* = 3 independent experimental replicates. Differences between the groups were examined using one-way ANOVA, and multiple comparisons were evaluated by Tukey HSD test. Different letters indicate statistically significant differences (*p* < 0.05).

Collagen and elastin are the main components of the ECM, essential for maintaining skin elasticity and resilience. Elastase and collagenase can degrade elastin and collagen, respectively, and their degradation in cells promotes skin aging. Therefore, inhibiting the activity of these two proteases in cells can alleviate skin aging. This study used EGCG as a positive control to test the inhibitory effects of SFRP and SFEP on collagenase and elastase. The polyphenol extract from *S. fusiforme* showed good inhibitory effects on collagenase (Figure 2a). At a polyphenol concentration of 10 μg/mL, the inhibition rates of SFRP and SFEP on collagenase were 87.12% and 64.2%, respectively. At a concentration of 100 μg/mL, the inhibition rates were 93.44% and 95.72%, comparable to the positive control EGCG (93.69%). As shown in Figure 2b, both SFRP and SFEP have inhibitory effects on elastase, with SFRP showing stronger inhibition. The inhibition rate of 100 μg/mL SFRP on elastase (99.35%) was higher than that of the control group EGCG (73.11%).

### 3.4. Inhibitory Effects of S. fusiforme Polyphenol Extracts on the Activity of mTYR

#### 3.4.1. Inhibition of mTYR Activity by SFRP and SFEP

**Figure 3:**
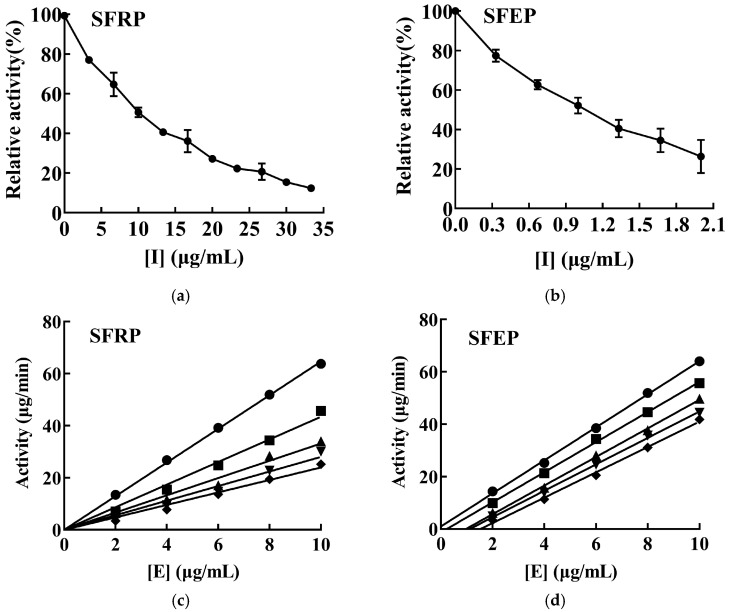
Inhibitory effects and mechanism of SFRP and SFEP on mTYR activities. Inhibition of mTYR catalytic activity by SFRP (**a**) and SFEP (**b**) using L-DOPA as reaction substrate, and *n* = 3 experimental replicates. In the study of the inhibition mechanism, the enzyme concentration was varied and the substrate L-DOPA concentration was controlled to be constant, and the enzyme activity of mTYR was determined at different concentrations of SFRP (**c**) and SFEP (**d**), respectively. The final concentrations of SFRP were 0 (●), 4 (■), 8 (▲), 12 (▼), and 16 (♦) μg/mL in (**c**). The final concentrations of SFEP were 0 (●), 0.8 (■), 1.6 (▲), 2.4 (▼), and 3.2 (♦) μg/mL in (**d**).

The inhibitory effects of SFRP and SFEP on mTYR activity were investigated using L-DOPA as the substrate, as depicted in Figure 3a,b. The activity of mTYR decreased gradually with increasing concentrations of the effectors, demonstrating concentration dependence. The IC50 values for the inhibition of mTYR activity by SFRP and SFEP were 9.89 and 0.99 μg/mL, respectively.

#### 3.4.2. Inhibitory Mechanism of SFRP and SFEP on mTYR

The inhibitory mechanisms of SFRP and SFEP on mTYR activity were investigated by varying the enzyme concentration while maintaining a constant substrate L-DOPA concentration, and measuring the enzyme activity of mTYR at different concentrations of SFRP and SFEP. SFRP produced a set of straight lines passing through the origin (Figure 3c), with slopes decreasing as the polyphenol concentration increased, indicating that the inhibition mechanism of SFRP on mTYR activity is reversible. This means SFRP interacts with the enzyme through non-covalent bonds, reducing the oxidation rate of the substrate L-DOPA by mTYR. These non-covalent bonds can be removed by physical methods, and thus, SFRP does not cause permanent denaturation or inactivation of the enzyme. Conversely, SFEP resulted in a set of parallel lines (Figure 3d), suggesting that SFEP does not affect the rate of the enzymatic reaction, indicating an irreversible inhibition of mTYR activity by SFEP. SFEP binds to the enzyme through covalent bonds, permanently inactivating it and reducing the effective enzyme concentration.

#### 3.4.3. Inhibition Type of mTYR Activity by SFRP

**Figure 4:**
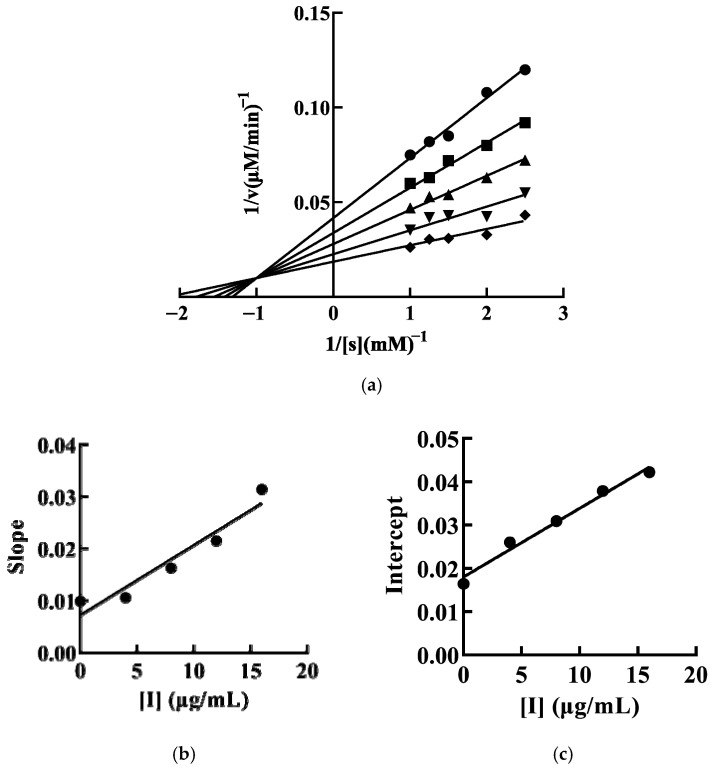
The inhibitory type of SFRP on mTYR. The final concentrations of SFRP were 0 (●), 4 (■), 8 (▲), 12 (▼), and 16 (♦) μg/mL. The inhibition of mTYR activity by SFRP was plotted by the double inverse method by controlling the enzyme constant value and varying the substrate concentration (**a**). The SFRP concentration was plotted quadratically by double inverse slope (**b**) and vertical axis intercept (**c**), respectively.

Reversible inhibition is classified into four types: competitive, noncompetitive, uncompetitive, and mixed. We have determined that SFRP inhibits mTYR activity through a reversible mechanism. Therefore, we further investigated the type of inhibition exerted by SFRP on mTYR activity. Keeping the enzyme concentration constant while varying the substrate concentration, a double reciprocal plot of 1/v against 1/[S] was constructed. The inhibition of mTYR activity by SFRP, as demonstrated by the double reciprocal plot, consists of a set of lines intersecting in the second quadrant. With increasing concentrations of SFRP, the maximum reaction velocity of the enzyme-catalyzed reaction decreases, and the Michaelis constant (Km) increases with the concentration of SFRP, indicating that the inhibition of mTYR activity by SFRP is of the mixed type (Figure 4a). Secondary plots of the slopes (Figure 4b) and vertical intercepts (Figure 4c) against SFRP concentration yield inhibition constants for the enzyme–substrate complex (KIS) and the free enzyme (KI) of 11.25 μg/mL and 5.54 μg/mL, respectively. The value of KIS is greater than that of KI, suggesting that SFRP has a higher affinity for the free enzyme than for the enzyme–substrate complex.

### 3.5. Effects of SFRP on Melanogenesis and TYR Activity in B16F10 Cells

Comparing the in vitro antioxidant, anti-aging, and whitening activities of SFRP and SFEP, and considering the use of non-toxicizing extraction methods, we have selected SFRP as the main target for further studies on whitening activity.

**Figure 5:**
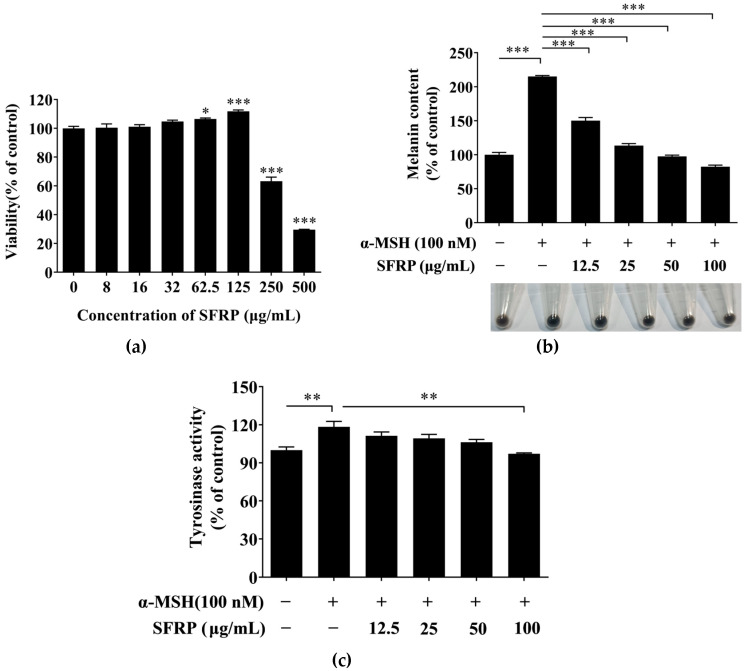
Inhibitory activity of SFRP on melanogenesis in B16F10 cells. Effects of SFRP on proliferative activity (**a**), melanin content (**b**) and TYR activity (**c**). Data are expressed as the mean ± SD from *n* = 3 independent experimental replicates. The asterisks denote significant differences, * *p* < 0.05, ** *p* < 0.01, *** *p* < 0.001, by one-way ANOVA followed by LSD post hoc test.

B16F10 cells were treated with various concentrations of SFRP (0–500 μg/mL) for 24 h to measure the cytotoxicity of SFRP. SFRP at concentrations of 8–125 μg/mL had no toxic effect on cell viability compared with the control group (Figure 5a), and while the SFRP concentration reached 250 μg/mL, the viability of B16F10 cells significantly decreased (*p* < 0.001). Therefore, 8–125 μg/mL SFRP was used in the next experiment.

To estimate the inhibitory effect of SFRP on the process of melanogenesis in B16F10 cells, the content of melanin in B16F10 cells treatment with SFRP for 60 h was measured by alkaline lysis. As shown in Figure 5b, SFRP significantly decreased the melanin content in B16F10 cells induced by the α-MSH in a dose-dependent manner. Contrasted with the control, the content of melanin in the α-MSH stimulated-B16F10 cells was increased 215.18%; however, the content of melanin was decreased to 82.42% in B16F10 cells with the treatment of SFRP at 100 μg/mL compared with the control.

The content of melanin is directly related to the TYR activity. Thus, we further explore the cellular TYR activity in the α-MSH stimulated-B16F10 cells with different concentrations of SFRP. As shown in Figure 5c, the activity of cellular TYR in α-MSH-stimulated B16F10 cells was significantly increased (by 114.65%) compared with the control (*p* < 0.05). Compared to the positive group, 100 μg/mL SFRP significantly inhibited the intracellular TYR activity induced by α-MSH, which was decreased to 97.16% (*p* < 0.01).

### 3.6. Effects of SFRP on the Expression of Melanogenesis-Related Proteins

**Figure 6:**
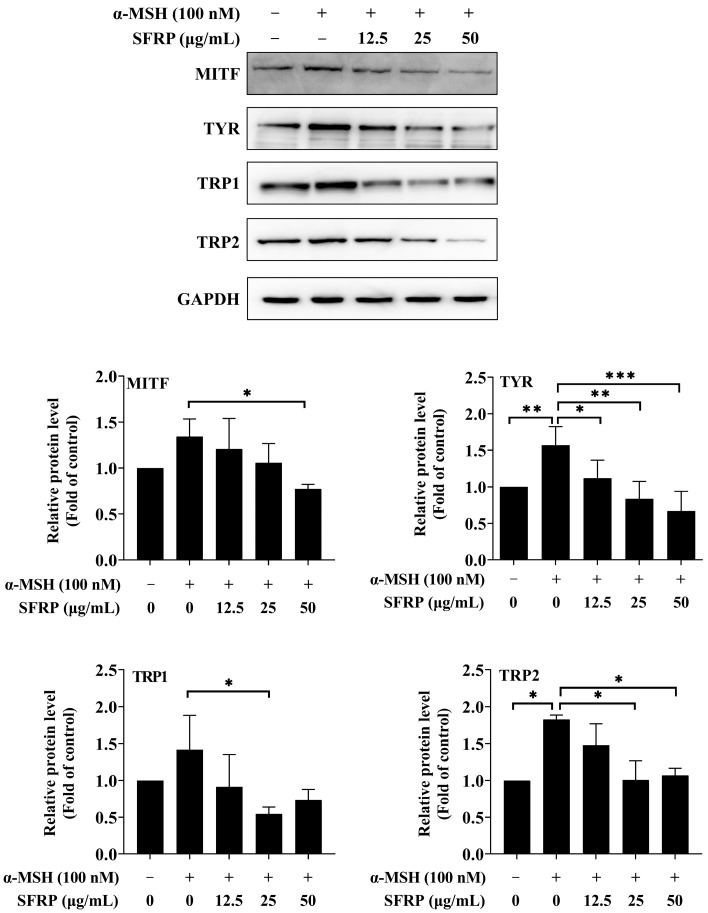
Effects of SFRP on the expressions of melanogenesis-related proteins in B16F10 cells. The intensity of the blotting bands was measured by Quantity One Software 4.6.2, and the results are shown as a bar graph representing the relative expression levels. Each bar represents the mean ± SD of *n* = 3 experimental replicates. The asterisks denote significant differences, * *p* < 0.05, ** *p* < 0.01, *** *p* < 0.001, by one-way ANOVA followed by LSD post hoc test.

The effects of SFRP on the expression of melanogenesis-related proteins are shown in Figure 6. The results showed that the expression of TYR, TRP1, and TRP2 in the cells was strongly increased by α-MSH compared to the control. SFRP could significantly attenuate the expression of all four proteins. The results suggested that SFRP relieved the hyperpigmentation induced by α-MSH in B16F10 cells through downregulating the expression of melanogenesis-related proteins.

### 3.7. Effects of SFRP on the Melanogenesis-Related Signaling Pathway

**Figure 7:**
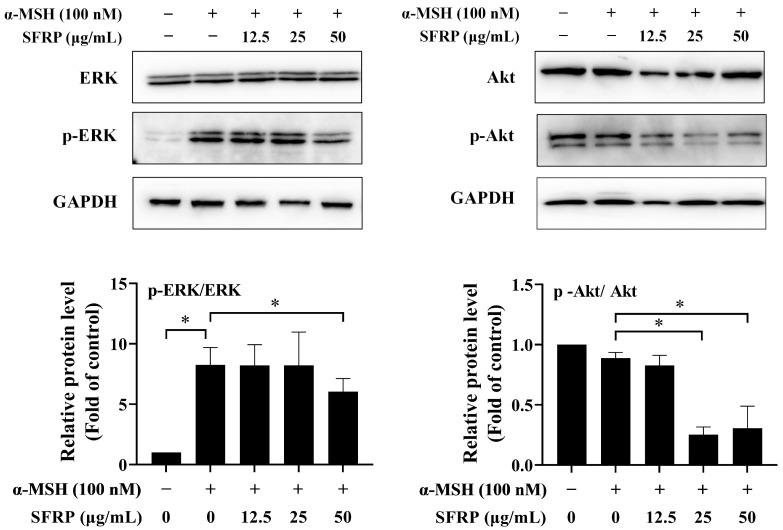
Effects of SFRP on ERK and Akt signaling pathway in α-MSH-stimulated cells. The intensities for phosphorylation levels of ERK and Akt were measured by Western blotting. The intensity of the blotting bands was measured by Quantity One Software 4.6.2, and the results are shown as a bar graph representing the relative expression levels. Each bar represents the mean ± SD of *n* = 2 experimental replicates. The asterisks denote significant differences, *p* < 0.05, by one-way ANOVA followed by LSD post hoc test.

To better understand the molecular mechanisms contributing to the anti-melanogenesis effects of SFRP, we further investigated the expression of p-ERK/ERK and p-Akt/Akt. The results showed that SFRP reduced the expression of p-ERK or p-Akt at concentrations of 50 μg/mL and 25–50 μg/mL, respectively, while the total amount of ERK and Akt proteins remained almost unchanged (Figure 7). These results suggested that SFRP might inhibit melanin synthesis in α-MSH-stimulated B16F10 cells by downregulation of melanogenesis regulators through the PI3K/Akt and ERK signaling pathway.

### 3.8. Evaluating the Skin Toxicity of SFRP

**Figure 8:**
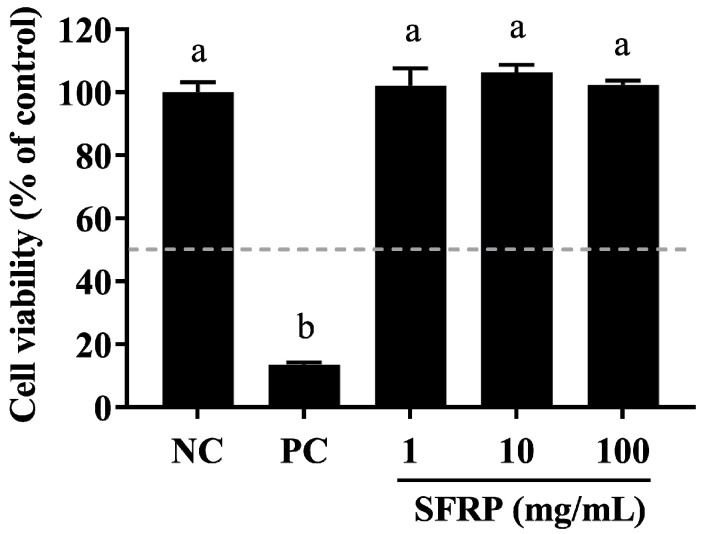
Assessing the cell viability of the EpiSkin™ 3D human skin model during skin irritation testing. Epidermis units were exposed to 10 μL of SFRP at concentrations of 1, 10, and 100 mg/mL, with 5% SDS (as a positive control, PC) or H_2_O (as a negative control, NC) for 15 min. After washing, they were moved into fresh medium and incubated for 42 h. Cell viability was then measured using the MTT assay. Differences between the groups were examined using one-way ANOVA, and multiple comparisons were evaluated by Tukey HSD test. Different letters indicate statistically significant differences (*p* < 0.05).

In the EpiSkin™-based in vitro skin irritation assay, cell viability was measured at 13.57% following treatment with 5% SDS. Notably, there was no observed reduction in epidermal tissue activity following exposure to 1, 10, or 100 mg/mL of SFPR when compared to the negative control (Figure 8). Adhering to the guidelines of OECD TG439, all formulations of SFPR were classified as non-irritants to human skin, as the cell viabilities remained above 50% after a 15 min exposure and a subsequent 42 h incubation period.

## 4. Discussion

Over the past few years, researchers have mainly focused on plant-derived natural compounds in the screening of agents resistant to melanin generation. The results showed that some compounds with antioxidant activity, such as α-tocopherol and ascorbic acid derivatives, played an important role in inhibiting melanin. This suggested that the antioxidant mechanism might be closely associated with pigmentation therapy [24,25,26]. Brown algae phenolic extracts are a kind of natural metabolites with various biological activities, and their antioxidant activity has been confirmed by many studies, suggesting that brown algae phenolic extracts exhibit the potential to inhibit melanin properties. In the present study, both SFRP and SFEP from *S. fusiforme* have strong antioxidant activity as well as TYR inhibitory activity in vitro, suggesting they have potential skin whitening properties and can be used to treat skin pigmentation disorders.

It has been proved that TYR plays an important role in the process of melanin biosynthesis, which catalyzes tyrosine hydroxylation to form DOPA and other intermediates [4]. TYR is a monooxygenase with a pair of copper ions in its active center [27]. TYR inhibitors improve the color of skin by inhibiting the formation of melanin precursors. Previous studies have found that polyphenols of brown algae compounds, with a large number of phenolic hydroxyl groups in the structure, form hydrogen bonds with the residues of TYR catalysis and allosteric sites, and chelate with copper ions in the active center of the enzyme, changing the conformation of TYR and inhibiting the catalytic activity of TYR [13]. Kim et al. demonstrated that 2-chlorophenol and 2-O-(2,4,6-trihydroxyphenyl)-6,6′-bieckol isolated from *E.cava* could dramatically inhibit TYR activity by blocking the entrance to the active site [28]. 974-A from *E.stolonifera* showed the potent competitive inhibitory of mushroom TYR by forming hydrogen bonds which reside at the catalytic and allosteric sites [13]. In the present study, both SFRP and SFEP could greatly inhibit the activity of mTYR activity in a dose-dependent manner.

The mechanism of inhibition of mTYR by SFRP and SFEP was reversible inhibition and irreversible inhibition, respectively. Reversible inhibitors, compared to irreversible counterparts, display reduced off-target toxicity and sensitization, minimize the risk associated with permanent protein modification, maintain their activity without inactivation, and provide prolonged effects within the catalytic system [29]. Compared to SFEP, SFRP exhibits superior free radical scavenging activity and inhibitory effects against elastase and collagenase. In terms of extraction methods, SFEP utilizes ethyl acetate extraction, whereas SFRP does not involve organic solvents, making it a more environmentally friendly and safer option. Consequently, this study focuses on SFRP for further investigation for inhibitory activity of melanogenesis in α-MSH-induced B16f10 cells. Our results indicated that SFRP significantly suppressed the melanin content and activity of TYR in cells in a concentration-dependent manner, which was consistent with the inhibition of 7-phloroeckol separated from *E. Cava* [30].

When the skin is exposed to the environment, the solar ultraviolet radiation stimulate keratinocytes to produce α-MSH. The α-MSH subsequently binds to melanocortin receptor 1 (MC1R) on the surface of melanocytes, and then activates intracellular adenylate cyclase to initiate a series of complex protease cascade reactions to regulate melanin synthesis [31]. In melanocytes, the production of melanin is controlled by at least three regulator proteins, TYR, TRP-1, and TRP-2 [32]. Our results demonstrated that SFRP significantly inhibited the expression of TYR, TRP-1, and TRP-2 in α-MSH-induced B16F10 cell. There is extensive evidence demonstrating that the master regulator of MITF transcriptionally regulates the expression of TYR, TRP-1, and TRP-2. In the present study, the reduction in TYR, TRP-1, and TRP-2 might be regulated by the downregulation of MITF. These findings align with prior studies demonstrating that purified phlorotannins, such as Diphlorethohydroxycarmalol derived from *Ishige okamurae* [33] and Phlorofucofuroeckol-A isolated from *Ecklonia cava* [34], similarly downregulate the expression of melanogenic proteins.

Several signaling pathways regulate melanin biosynthesis, including the cyclic adenosine monophosphate (cAMP) signaling, mitogen-activated protein kinase (MAPK) signaling, phosphatidylinositol 3-kinase (PI3K)/AKT signaling, and Wnt/β-catenin signaling pathways [35]. In the present study, a significant decrease in both p-ERK/ERK and p-Akt/Akt in B16F10 cells after SFRP incubation was investigated. The MAPK/ERK signaling pathway is triggered by the activation of the c-Kit receptor combined with the stem cell factor (SCF), primarily secreted by keratinocytes in response to external stimulation. Subsequently, ERK is phosphorylated and transferred into the nucleus to phosphorylate the MITF at the Ser73 site, which leads to the degradation of phosphorylated MITF by the ubiquitin-dependent proteasome [36]. Kim et al. also demonstrated that octaphlorethol A, isolated from *Ishige foliacea*, can inhibit the production of melanin in α-MSH-induced B16F10 cells with downregulation of MITF, TRP1, TRP2, and TYR through activating the ERK signaling pathway [37].

The PI3K/AKT signaling pathway is initiated by the activation of PI3K under the interaction of SCF/c-Kit or stimulation of cAMP. After PI3K is activated, Akt is phosphorylated at the Ser473 and Thr308 sites. However, the role of AKT in the melanin activation pathway is controversial. For instance, Yin et al. [38] found that flavonoids promote melanogenesis by upregulating the level of p-AKT in B16F10 cells. However, other studies have shown that sesamol can inhibit melanin production in B16F10 cells by upregulating p-AKT [39]. Research indicates that the phosphorylation of AKT leads to the phosphorylation of GSK-3β at ser21/9, which reduces the enzymatic activity of GSK-3β. This inhibits the ubiquitination and degradation of β-catenin (p-β-cateninser33/37), allowing β-catenin to accumulate intracellularly and subsequently enter the nucleus to promote MITF transcription [38,40]. Additionally, it has been reported that unphosphorylated GSK-3β can phosphorylate MITF at Ser298, increasing the localization of MITF-M to the M-box in the TYR promoter, thereby inducing melanogenesis. Therefore, the phosphorylation of GSK-3β at Ser21/9 and inactivation of GSK-3β will lead to reduced phosphorylation levels of MITF Ser298, thereby inhibiting the binding of MITF-M to the M-box and reducing the expression of TYR and TYRP-1 [41,42,43]. Meanwhile, AKT can also induce the phosphorylation of MITF at Ser409, promoting the destabilization and degradation of MITF [44]. Lee et al. demonstrated that dioxinodehydroeckol isolated from *Ecklonia stolonifera* reduced melanin synthesis by regulating the MITF signaling pathway through upregulation of Akt phosphorylation [14]. However, in the present study, we found that AKT phosphorylation was significantly inhibited by SFRP, suggesting that the reduction in melanin content in B16F10 cells caused by SFRP may be associated with the inactivation of GSK-3β and the accumulation of β-catenin.

Currently, *S. fusiforme* extract is internationally recognized as a safe and effective cosmetic ingredient (INCI name: *Hizikia fusiforme* EXTRACT) and is widely used in numerous cosmetic products. The natural origin of *S. fusiforme* polyphenol extracts, their demonstrated safety, and the well-defined melanin inhibition mechanisms, particularly those of SFRP, make them an excellent supplement to natural cosmetic ingredients and an attractive alternative to synthetic compounds. Additionally, these findings position SFRP as a promising component for the development of next-generation functional food products. However, regarding all the *S. fusiforme* extracts, their application in functional foods and nutraceuticals remains limited. Despite their long-standing use in traditional medicine and food, and the promising preclinical results demonstrating their broad bioactivities, current clinical applications are still constrained. Future research should focus on further investigating the oral safety and efficacy of *S. fusiforme* polyphenol extracts, aiming to provide consumers with safer and more effective solutions for skin whitening, anti-aging, and overall skin health improvement.

## 5. Conclusions

In this study, we obtained different polyphenol extracts from *S. fusiforme* through macroporous resin and ethyl acetate extraction. We compared their antioxidant, anti-wrinkle, and TYR inhibitory activities, and ultimately explored the melanogenesis inhibitory mechanism of SFRP at the cellular level. The result suggests that the anti-melanogenesis effects of SFRP are mediated by the downregulation of TRY, TRP1, and TRP2 protein expression through MITF-dependent transcriptional regulators, which is associated with the suppression of the PI3K/Akt and MAPK/ERK signaling pathways. These findings underscore the untapped potential of polyphenols from *S. fusiforme* in addressing skin pigmentation disorders and aging concerns. By demonstrating the antioxidant and anti-aging properties of SFRP and SFEP, this research opens new avenues for the development of natural, safe, and effective cosmetic ingredients. Moreover, the elucidation of the mechanisms of SFRP provided valuable insights into the regulation of melanogenesis and offers targets for further exploration in skin biology research. Overall, this research contributes to the growing body of evidence supporting the use of brown algae extracts in nutraceuticals and cosmeceuticals.

## Data Availability

The original contributions presented in the study are included in the article, further inquiries can be directed to the corresponding author.
